# Renal Denervation as a Complementary Treatment Option for Uncontrolled Arterial Hypertension: A Situation Assessment

**DOI:** 10.3390/jcm12175634

**Published:** 2023-08-29

**Authors:** Max Wagener, Eamon Dolan, Samer Arnous, Joseph Galvin, Andrew W. Murphy, Ivan Casserly, Joseph Eustace, Stephen O’Connor, Charles McCreery, James Shand, Catherine Wall, Saijad Matiullah, Faisal Sharif

**Affiliations:** 1 University Hospital Galway, University of Galway, H91 TK33 Galway, Ireland; 2 Stroke and Hypertension Unit, Connolly Hospital, D15 X40D Dublin, Ireland; 3 University Hospital Limerick, V94 F858 Limerick, Ireland; 4 The Mater Misericordiae University Hospital, D07 R2WY Dublin, Ireland; 5 Turloughmore Medical Centre, University of Galway, H91 TK33 Galway, Ireland; 6 University College Cork, T12 K8AF Cork, Ireland; 7 St. James’s Hospital, D08 NHY1 Dublin, Ireland; 8 St. Vincent’s University Hospital, D04 T6F4 Dublin, Ireland; 9 Tallaght University Hospital, D24 NR0A Dublin, Ireland; 10 University Hospital Waterford, X91 ER8E Waterford, Ireland

**Keywords:** renal denervation, arterial hypertension, resistant hypertension, uncontrolled hypertension

## Abstract

Uncontrolled arterial hypertension is a major global health issue. Catheter-based renal denervation has shown to lower blood pressure in sham-controlled trials and represents a device-based, complementary treatment option for hypertension. In this situation assessment, the authors, who are practicing experts in hypertension, nephrology, general practice and cardiology in the Republic of Ireland, discuss the current evidence base for the BP-lowering efficacy and safety of catheter-based renal denervation with different modalities. Although important questions remain regarding the identification of responders, and long-term efficacy and safety of the intervention, renal denervation has the potential to provide much-needed help to address hypertension and its adverse consequences. The therapeutic approach needs to be multidisciplinary and personalised to take into account the perspective of patients and healthcare professionals in a shared decision-making process.

## 1. Introduction

Uncontrolled arterial hypertension is a global health issue of enormous dimensions. Pooled data analysis from 2019, shows a global age-standardised prevalence of hypertension in adults (30–79 y) of 32% (95% credible interval (CrI) 30–34) in women and 34% (95% CrI 32–37) in men [1]. As a major driver for cardiovascular (CV) disease and associated morbidity and mortality, early preventive measures to control elevated blood pressure (BP) are mandatory [2]. In hypertensive patients, every reduction in systolic BP by 10 mmHg significantly reduces major CV events (MACE) by 20% (relative risk (RR) of 0.80 (95% confidence interval (CI) 0.77–0.83)) and all-cause mortality by 13% (RR 0.87, 95% CI 0.84–0.91) [3]. Furthermore, a reduction of systolic BP of 10 mmHg has proven to be effective in reducing coronary heart disease (RR 0.83, 95% CI 0.78–0.88), stroke rates (RR 0.73, 95% CI 0.68–0.77) and heart failure (RR 0.72, 95% CI 0.67–0.78) [3].

Despite the high prevalence and proven benefit of BP control, control rates are poor, being only 23% (95% CrI 20–27) in female and 18% (95% CrI 16–21) in male patients [1]. A multiplicity of factors contribute to a lack of control, e.g., physician inertia, patient non-adherence, lack of social support, depression and the complexity of polypharmacy regimens in more severe cases of hypertension [4,5]. In general, up to 40% of patients included in medication trials are either non-adherent or have reduced drug-exposure due to irregular intake of their medication [5].

Based on the World Health Organisation CV mortality rates, Ireland is a moderate CV risk country [6,7]. Depending on CV risk (SCORE2, SCORE2-OP) and baseline BP, current guidelines recommend a stepwise approach with lifestyle changes and pharmacotherapy, aiming to lower BP <140/90 mmHg in all patients and systolic BP (SBP) to 120–130 mmHg in most patients (aged 18–69 years) [6,8].

In the last few years, evidence from a number of high-quality clinical trials has provided strong support for the BP-lowering efficacy and safety of catheter-based renal denervation (RDN) using radiofrequency (RF) energy or ultrasound-based ablation. This has positioned the intervention as a highly promising complementary tool in the treatment of hypertension [9,10,11].

As discussed in greater detail below, these recent trials offer reassurance about the efficacy of RDN. The re-emergence of the intervention as an important novel treatment has led to the publication of a number of position statements and consensus documents [4,12,13,14,15,16]. Given the time lag between the advent of new clinical data and updates to guidelines, such consensus documents are valuable to assess the state of the art and allow for early adoption of the intervention in current and future standards of care. 

The current document presents the position of the authors, who are practicing experts in hypertension, nephrology, general practice and cardiology in the Republic of Ireland. It is intended as a situation assessment in the Irish context and does not aspire to the status of a formal guideline. It is intended to offer an overview on the rationale for the use of RDN in selected patients.

## 2. Rationale for Renal Denervation in Hypertension

**Figure 1:**
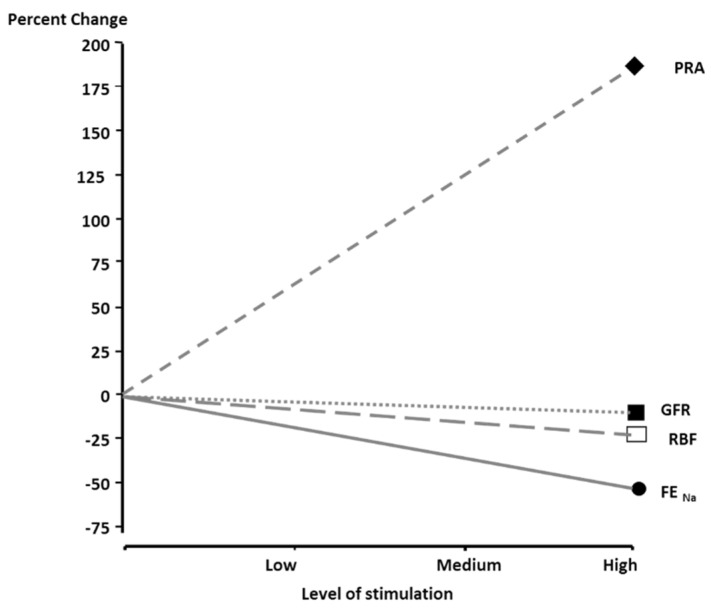
Effects of renal nerve stimulation on plasma renin activity (PRA), glomerular filtration rate (GFR), renal blood flow (RBF) and fractional excretion of sodium (FE_NA_). From Johns et al. (Compr Physiol 2011;1:731–767) [21].

The rationale for RDN is to attenuate the crosstalk between the kidney and the central nervous system [17]. The renal sympathetic nerves play an important role in homeostasis and regulation of BP. Renal sympathetic nerve stimulation is associated with increased plasma renin activity (PRA), renal tubular sodium reabsorption and decreased urinary sodium excretion and slightly reduced renal blood flow Figure 1 [18]. Renal sympathetic drive is elevated in many patients with primary and secondary hypertension as well as in obesity-related hypertension [19,20]. Conversely, ablation of renal nerves has shown in experimental models to reduce efferent renal sympathetic activity, prevent the development of hypertension and reduce BP [18].

## 3. Renal Denervation—State of the Evidence

### 3.1. Efficacy

**Figure 2:**
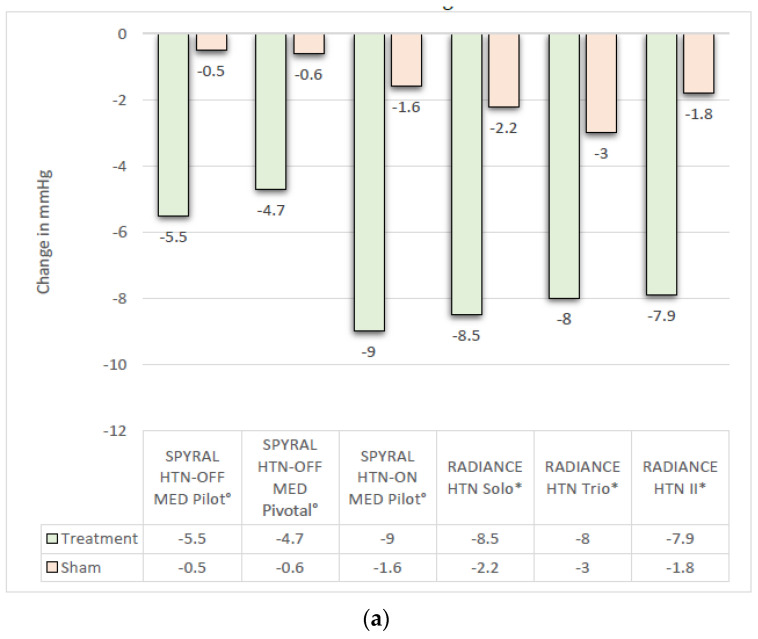

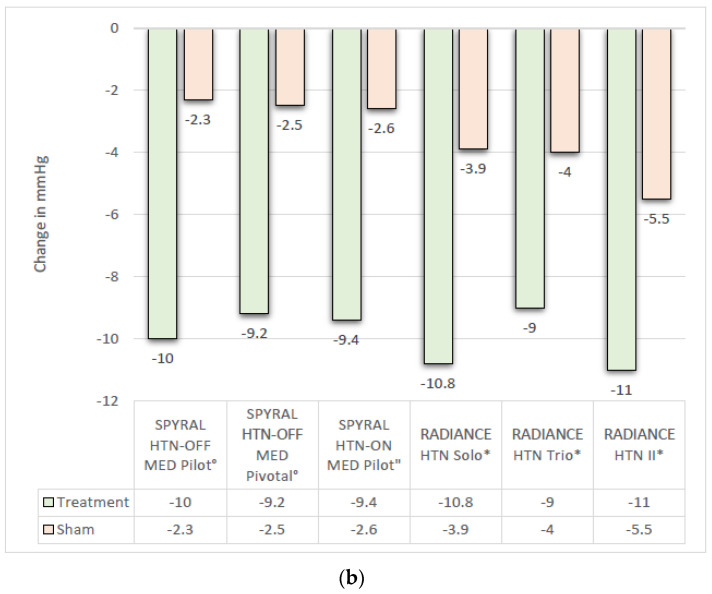
(**a**) Change in SBP as assessed by ABPM. Data represented by trial-defined primary efficacy endpoints. (°) Change in **24 h** systolic BP as assessed by ABPM. (*) Change in **daytime** systolic BP as assessed by ABPM. (**b**) Change in systolic **office BP** from baseline to trial-defined primary endpoint time at 2 (*), 3 (°) and 6 (“) months.

Between 2017 and 2023, six high-quality sham-controlled randomised clinical trials of RDN were completed, using RF (Symplicity Spyral™ catheter, Medtronic, Dublin, Ireland) or ultrasound (Paradise™ system, ReCor Medical, Palo Alto, CA, USA) [9,22,23,24,25,26,27]. All randomised controlled trials (RCTs) demonstrated a significant and clinically meaningful BP lowering effect (Figure 2a,b) both in the presence and absence of medication, and have shown a good safety profile for the interventions [9,11,22,23,25,27]. 

Considering the design flaws of the early trials, with neutral findings in the context of discrepancies of medication burden and incomplete ablations (see Section 3.2. below), second generation devices and new trial designs were introduced to reduce the effect of potential confounders. In addition to the randomised, sham-controlled trial design, the efficacy of RDN was analysed in absence (“off-med”) and presence (“on-med”) of antihypertensive medication [23,24,25,26]. Methodologically, a new BAYESIAN design was applied, being more stable to the effect of outliers than the previously used ANCOVA models and allowing for prespecified interim analysis [28,29].

Out of a range of devices, to date, three are still available, and their application has been evaluated in published trials and registries. Starting with the RF-based ablation (Symplicity Spyral™), the pilot and pivotal OFF-MED trials showed a significant reduction in 24 h SBP (24 h-SBP) in the treatment group in comparison to the sham control group of −5.5 (95%CI: −9.1 to −2.0) vs. −0.5 mmHg (95%CI: −3.9 to 2.90), respectively, *p* = 0.0414 (SPYRAL HTN-OFF MED, PILOT) and −4.7 (95%CI: −6.4 to −2.9) vs. −0.6 mmHg (95%CI: −2.1 to 0.9), *p* = 0.0005 (SPYRAL HTN-OFF MED PIVOTAL) [9,23]. These results were confirmed in the presence of antihypertensive medication (SPYRAL HTN-ON MED) with yet again a significant reduction in 24 h-SBP at 6 months, −9.0 (95%CI: −12.7 to −5.3) in the intervention group vs. −1.6 mmHg (95%CI: −5.2 to 2.0) in the sham group, *p* = 0.006 [22,26]. The final results of the expansion trial (NCT02439775) are pending and interim analysis as presented at ACC 2022 are discussed below. 

The second modality under investigation is the ultrasound-based Paradise™ System. In absence of antihypertensive medication, the significant effect of ultrasound-based RDN (RADIANCE HTN Solo) was proven, and a reduction in daytime SBP as assessed by ambulatory BP monitoring (ABPM) of −8.5 (±9.3) mmHg in the intervention group vs. −2.2 (±10.0) mmHg in the sham-group (*p* for difference = 0.0001) was achieved at 2 months follow-up [11]. Similar to the RF-based trial results, also in presence of antihypertensive drugs, the ultrasound-based RDN (RADIANCE HTN Trio) has proven to be effective with a reduction of daytime SBP (ABPM) by −8.0 (IQR −16.4, 0.0) mmHg in the intervention group vs. −3.0 mmHg (IQR−10.3, 1.8), *p* = 0.022, at 2 months follow-up [25]. The latest analysis on patients undergoing ultrasound-based RDN (RADIANCE II) confirmed the results of the preceding trials and patients undergoing RDN had significantly lower daytime SBP at 2 months follow-up, −7.9 (±11.6) mmHg vs. −1.8 (±9.5) mmHg (*p* for difference <0.001) [30]. Patient-level pooled data analysis of the three RADIANCE trials confirmed the primary efficacy endpoint with a significant reduction in ambulatory 24 h-SBP and office SBP in the RDN group in comparison to the sham group [31]. After primary analysis, 6 months of data from the RADIANCE-HTN Solo trial showed similar BP-lowering effects with fewer medications in the RDN group than in sham-controlled patients [32]. Moreover, significantly (*p* = 0.008) more patients treated with RDN than with sham had no need for antihypertensive drugs at 6 months [32]. It remains unclear at present whether this will remain the case after prolonged follow-up [32].

Figure 2a,b summarises the reductions in SBP from baseline to the trial-defined endpoint times in the individual RCTs. The trial-specific BP cut-offs used for inclusion in the trials as well as the exclusion criteria at the kidney level are summarised in the Supplementary Table S1.

The third modality, alcohol-mediated RDN, is currently investigated in a number of clinical trials, (TARGET BP-OFF, NCT03503773, TARGET BP I, NCT02910414) and the results are pending [10,33,34]. 

As a significant reduction in BP was shown in randomised, sham-controlled trials using RF- and ultrasound-based modalities, a treatment class effect for RDN may be hypothesised, but the impact of different technologies on the rate of procedural success is yet to be analysed and data from other modalities are pending [25,26]. 

Whereas antihypertensive medication has fluctuating levels around the therapeutic range, RDN has the benefit of having an “always-on” acting profile [9,22,23,35,36]. Taking into account the prognostic value of night-time BP control, RDN may have an advantage over medication only [27,37]. Furthermore, uncontrolled BP and associated BP variability have been described as a surrogate for worse CV outcomes [38].

### 3.2. Neutral Trials

A few trials did not meet their primary efficacy endpoint. The intervention was largely written off by the clinical community and device industry in 2014, when the sham-controlled SYMPLICITY HTN-3 trial failed to show a significant BP reduction in the intervention group in comparison to the control group [39]. The results came as a considerable surprise, after the publication of several highly promising proof-of-concept and randomised, open-label studies [40,41,42,43,44,45,46]. The lack of effect in the SYMPLICITY HTN-3 trial could be attributed to a number of factors, importantly procedural technique and incomplete denervation, high rate of drug changes and lack of adherence to treatment [47,48]. Post hoc analysis showed that in non-African Americans, patients undergoing RDN had a significant reduction in office SBP at 6 months in comparison to the sham group (*p* = 0.01) [47]. In contrast in the African American population, the sham group had an unexpectedly lower office SBP at 6 months than the RDN group [47]. This finding was indicative of the confounding influence of medication changes (39% of patients had medication changes from baseline), and the use of vasodilators, which were more frequently prescribed in this subgroup [47].

The REDUCE HTN:REINFORCE trial used a balloon-based bipolar RF-based system [49]. This trial was terminated for apparent futility during recruitment and the predefined 8-week primary efficacy endpoint of 8-week change in 24-h ambulatory SBP was not met [49]. Nevertheless, a significant reduction in daytime and systolic office BP at 6 months in the RDN group may be indicative of delayed treatment effects [49].

To assess the efficacy of ultrasound-based RDN in a population of Asian ethnicity, with different hypertensive phenotypes, the REQUIRE trial was a randomised controlled trial in a Japanese and South Korean population [50]. This trial did not meet its primary endpoint and there was no difference in ambulatory BP at 3 months [50]. Potential confounders in this trial were non-standardised medication and lack of control of medication adherence and dosing, as suggested by the unexpectedly high treatment effect in the sham-group, as well as the inclusion of patients with presumed hyperaldosteronism [51]. The influence of ethnicity in the findings of the REQUIRE trial remains difficult to assess [50].

Of note, data of 337 patients from the SPYRAL HTN-ON MED Expansion Study [NCT02439775] presented at the ACC in November 2022 showed no significant difference in 24 h ambulatory BP in the RDN-arm in comparison to the sham control-arm. But the absolute reduction in BP by RDN was consistent with the previously published data on RF-based RDN and office BP was still significantly lower in the RDN-arm. The fact that patients in the sham-control arm had a significantly higher medication burden and part of the 24 h ABPM was assessed during the COVID-19 pandemic relativises the unmet primary endpoint [52]. 

These difficulties show the challenges associated with clinical trials of an interventional therapy in hypertensive patients.

### 3.3. Safety of Renal Denervation Procedures

Within the limits of current clinical experience with the technique, available data indicate that RDN has a very favourable safety profile. By design of the interventional therapy, mainly three safety aspects are to be discussed. 

First, the risk of vascular access complications. In the randomised controlled SPYRAL trials, no vascular access site complications were reported [9,22,23]. Pooled data analysis of the RADIANCE trials (*n* = 506) reported a single pseudoaneurysm that had to be treated with IV thrombin injections [31].

Second, contrast-induced kidney injury. None of the above mentioned RCTs reported a significant change in creatinine from baseline to follow-up or major contrast-induced kidney injury [9,22,23,31]. One transient doubling of plasma creatine in the RDN group was reported in the Radiance HTN-Trio trial and was associated with spironolactone use and resolved after stopping the latter [25]. Although over the course of three years, renal function declined more strongly in patients without (eGFR ≥ 60 mL/min/1.73 m^2^, reduction by 7.1  ±  16.7 mL/min/1.73 m^2^)  than with renal dysfunction (eGFR < 60 mL/min/1.73 m^2^, reduction by 3.7 (±16.2) mL/min/1.73 m^2^), both values lie within the range of the expected decline in renal function of patients with arterial hypertension [53]. A meta-analysis, including eGFR measurements from 2381 patients, found no clinical significant change in eGFR 9 months after RDN [54]. Of note, for safety reasons, eGFR < 40–45 mL/min per 1.73 m^2^ was an exclusion criterion in all of the sham-controlled RCTs and the assessed safety profile cannot be generalised to patients with a higher degree of kidney dysfunction. In the ongoing SPYRAL AFFIRM global study (NTC05198674), an eGFR of <30 mL/min/1.73 m^2^ was chosen as an exclusion cut-off, thus allowing the collection of efficacy and safety endpoints in patients with a higher degree of renal dysfunction.

Third, injuries at the kidney level such as renal artery stenosis, parenchymal lesions, embolic events and organ tissue damage. Acute risks include micro injuries to the renal arteries, which have been documented using intravenous ultrasound and optical coherence tomography [55]. No clinically significant sequelae have thus far been reported from these injuries. Procedure-related endothelial damage recovers within 7 days and injuries to the arterial wall and soft tissue appear to heal within 180 days [56,57]. In the RCTs, only one significant renal artery stenosis was reported in the RDN arm of the RADIANCE HTN Solo trial, although after independent review of the pre-interventional MR-angiography and selective angiography, the stenosis to be treated was retrospectively adjudicated to be pre-existing and not procedure-related [11]. Pooled data meta-analysis of the RDN trials including over 5000 patients showed an annual incidence rate of renal artery damage necessitating renal artery stenting of 0.20% per year (95% CI 0.12–0.29) [58]. In the context of the natural prevalence of renal artery stenosis in patients with hypertension (14.1% (95% CI 12.7–15.8%), pooled prevalence in a meta-analysis by de Mast et al.), and natural progression of disease if untreated, the risk of RDN-related renal artery damage seems low [59,60,61,62].

Although procedures were done with a first generation device (SYMPLICITY Flex ™), long-term results from the SYMPLICITY HTN-3 Trial did not show a significant difference in the composite safety endpoint (death from any cause, end-stage renal disease, an embolic event resulting in end-organ damage, renal-artery or other vascular complications, hypertensive crisis, or new renal-artery stenosis of more than 70%) at 36 months across the three groups (RDN, cross-over, non-cross-over) [63].

### 3.4. Influence of Ethnicity

Post hoc analysis of the SYMPLICITY HTN-3 trial showed a non-significant treatment response in the African American subpopulation. Although the potential influence of ethnicity cannot be ruled out, as discussed above, a major confounder seems to be the changes of medication in this subgroup (39% from baseline) [47]. To date, the data is suggestive of comparable safety and efficacy profiles for RDN across ethnicities [53,64,65]. In the Korean Global Simplicity Registry (GSR Korea), RF-based RDN led to a significant reduction in BP up to 36 months (*n* = 102), with 89.7% of participants achieving an office SBP reduction of ≥10 mmHg [64]. SYMPLICITY HTN-Japan reported similar results, with 86% of patients showing a significant reduction in office SBP of ≥10 mmHg at 36 months [65]. Further data acquisition and data analysis focusing on ethnicity are needed.

## 4. Long-Term Effects of Renal Denervation 

The sustainability of the antihypertensive effect of RDN is a relevant question with only limited data available at present. The best data available for long-term outcomes after RDN comes from the SYMPLICITY HTN-3 trial [63]. At 36 months, all included patients (*n* = 535) consisting of the original treatment group, and the original sham group with and without cross-over were analysed [63]. At 36 months, office SBP reduction was −26.5 mmHg (±25.9) in the RDN group and −5.7 mmHg (±24.4) in the sham group, (adjusted treatment difference −22.1 mm Hg (95% CI −27.2 to −17.0), *p* ≤ 0.0001). Also, 24 h-SBP was significantly lower in the RDN group at 36 months, −15.6 mmHg (±20.8) vs. −0.3 (±15.1) for the sham group (adjusted treatment difference −16.5 mm Hg (95% CI −20.5 to −12.5); *p* ≤ 0.0001). The treatment effect seemed to be progressive over time [63]. Yet again a progressive reduction in BP may be indicative of a delay in treatment effect after the procedure [63]. A non-conscious influence of more rigorously selected patients at the beginning of the trial cannot be excluded and the effect of unblinding and trial design in respect to antihypertensive medication must be considered [63]. Long-term analysis of the SPYRAL HTN-ON MED pilot (*n* = 80) showed a persisting significant treatment difference between both groups in favour of the RDN group at 36 months [26]. Showing a 24 h-SBP reduction of −18.7 mmHg (±12.4) in the RDN group, and −8.6 mmHg (±14.6) in the sham group (adjusted treatment difference −10 mmHg, (95% CI −16.6 to −3.3), *p* = 0.0039) [26]. In their latest trial, Kario et al. analysed circadian variability of BP after RDN [27]. In comparison to the sham-control arm, patients on three or more antihypertensive drugs undergoing RDN had significantly lower night and morning BP and higher BP control rates (SBP < 130 mmHg) at 36 months, 40% vs. 6%, *p* = 0.021 [27].

Medication burden may be a surrogate for long-term efficacy of RDN. In RADIANCE-HTN SOLO, the number of drugs needed to control BP at 12 months was significantly lower after RDN than after sham-procedure [24]. In the Global SYMPLICITY Registry, the largest currently followed cohort, there was no escalation in the burden of medication over 3 years with sustained and significant reductions in both office and ambulatory BP [53].

Limited data are available from follow-up of more than 3 years. A recent prospective single arm interventional study reported 48 months follow-up data of 49 patients treated for resistant hypertension [66]. Patients were on an average of six antihypertensive drugs and underwent RF-based RDN (Symplicity Flex™ or Spyral™) [66]. The mean reduction from baseline (±SD) in 24 hr ambulatory SBP at 48 months was 11 ± 25 mmHg (*p* < 0.001), while office SBP was reduced by 7 ± 23 mmHg (*p* < 0.02) [66].

## 5. Cardiovascular Outcomes after RDN

**Figure 3:**
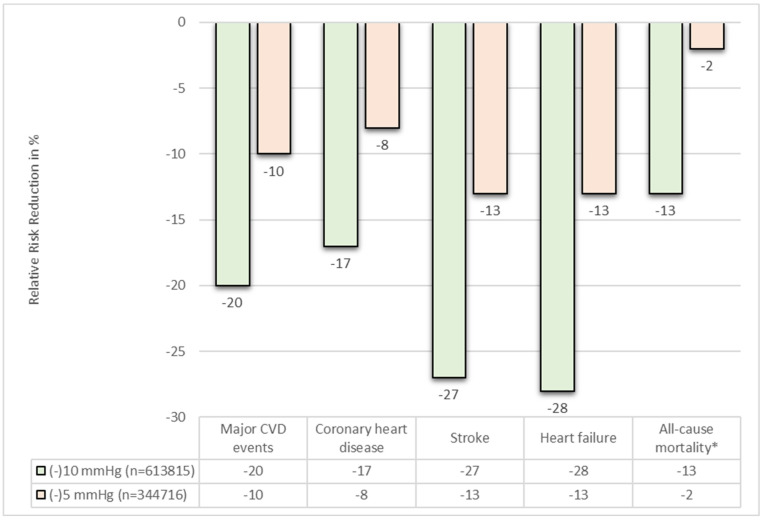
Relative Risk Reduction in adverse outcomes with sustained reductions in systolic BP by 5 (orange) and 10 (green) mmHg. Adapted from *Lancet. 2016;387(10022):957–967* [3] and *Lancet. 2021;397(10285):1625–1636* [67] (* reduction in all-cause mortality not significant (RR 0.98, 95%CI 0.96–1.01) for –5 mmHg reduction in SBP, but CV mortality significantly reduced RR 0.95 (95% CI 0.92–0.99).

Hypertension is a well-accepted surrogate marker of CV risk and long-term reductions in BP can be expected to reduce the risk of clinical outcomes [3]. Large scale meta-analysis of antihypertensive drug trials have shown that sustained reductions of SBP by 10 mmHg in hypertensive patients lead to a significant reduction in relative risk for MACE (RR per 10 mmHg reduction in SBP = 0.80, 95% CI 0.77–0.83), coronary heart disease (RR 0.83, 95% CI 0.78–0.88), stroke (RR 0.73, 95% CI 0.68–0.77) and heart failure (RR 0.72 (0.67–0.78) [3]. Although to a lesser extent, this significant outcome benefit remains true for a sustained SBP reduction by 5 mmHg [67]. These meta-analyses have also shown mortality benefit in patients with sustained BP reduction by 10 mmHg (all-cause mortality RR 0.87, 95% CI 0.84–0.91) and 5 mmHg (CV mortality RR 0.95, 95% CI 0.92–0.99), Figure 3 [3,67].

A probabilistic analysis based on the mean cohort changes in office SBP in the Global SYMPLICITY Registry indicated a significant reduction in MACE (composite endpoint of non-fatal stroke, non-fatal myocardial infarction and CV death) over 3 years after RDN MACE rate 8.6% (±0.7%) vs. 11.7% (±0.9%) in a projected control group without treatment, *p* < 0.01 [68]. This reduction in MACE was primarily due to a reduced stroke incidence (4.5% ± 0.5% actual vs. 6.9% ± 0.8 projected risk without treatment, *p* < 0.01) [68]. While no substitute for an outcome-driven trial, such analyses are in agreement with our current knowledge of the need to control BP in hypertensive individuals and underlines the potential role for RDN.

## 6. Cost-Effectiveness of Renal Denervation

The potential of long-term reduction of adverse CV events by reducing BP raises the question of cost-effectiveness of RDN as a one-in-a-lifetime procedure [3]. Early trial data was indicative of RDN being a cost-effective treatment option [69]. In patients with resistant hypertension, elevated 10-year CV risk, but without established CV disease, RDN is a cost-effective treatment option [70]. As incremental-cost-effectiveness-ratios (ICER) are specific to individual healthcare systems, no exact values can be mentioned here, but cost-effectiveness has been shown in different healthcare systems outside of Ireland [69,70,71].

## 7. Where Does Renal Denervation Fit in the Antihypertensive Treatment Toolkit?

The available data indicate that RDN has proven BP-lowering effects and a good safety profile. Although the handful of RCTs demonstrated antihypertensive effects in a heterogeneous patient population, the challenge to identify responders and non-responders to RDN remains. Real-life data from the Global SYMPLICITY Registry indicate a similar effect on BP levels from RDN among patients with varying CV risk and comorbidities [53,72].

The ‘always on’ effect of the intervention appears to produce more consistent BP reduction than pharmacotherapy even when the latter is taken regularly. Hence, it may be a particularly beneficial option in subjects with higher-than-expected nocturnal BP (non-dippers and reverse dippers) or those with a large early morning surge in BP, particularly in older people. Both conditions are associated with increased CV risk [73,74,75,76,77]. 

The ‘always on’ effect and once in a lifetime procedure is an advantageous feature of RDN for BP control, especially in the context of non-adherence to pharmacotherapy due to polypharmacy [5,78,79]. Adherence correlates inversely with the number of antihypertensive drugs taken, which is particularly important in high-risk patients [5,80,81].

As mentioned above, RDN has proven its efficacy and safety in patients with and without antihypertensive medication in resistant and/or uncontrolled arterial hypertension. Considering the achieved treatment effect by RDN, this interventional treatment seems to be a complementary tool to control BP alongside the state-of-the-art pharmacotherapy.

## 8. Consensus Points

The following consensus points have been elaborated based on clinical expertise and an extensive literature review by a multidisciplinary national advisory board. Specialists of the following disciplines are represented in this advisory board: **Cardiology:** Dr. M. Wagener, Dr. S. Arnous, Prof. J. Galvin, Prof. I. Casserly, Dr. S. O’Connor, Dr. C. McCreery, Dr. J. Shand, Dr. S. Mattiullah, Prof. F. Sharif; **Stroke and Hypertension:** Dr. E. Dolan; **General Practice**: Prof. A. W. Murphy; **Nephrology**: Prof. J. Eustace, Prof. C. Wall.

### 8.1. Point 1

*Insufficiently controlled BP is a large public health problem and greater control of BP is needed to reduce adverse outcomes.*

This point has been made for decades, and remains even more relevant today.

### 8.2. Point 2

*A multidisciplinary team should be involved in the diagnosis and treatment of resistant and uncontrolled hypertension*.

As has been noted by other experts, most patients with hypertension encountered in routine clinical care will not have difficult-to-treat, let alone resistant, hypertension. A multidisciplinary evaluation is necessary to exclude secondary causes, and identify possible hypertension-mediated organ damage or severe comorbidities, which would increase the need and urgency to reduce BP. We encourage the setting up of multidisciplinary refractory hypertension clinics with input from cardiology, endocrinology, nephrology and clinical pharmacists.

### 8.3. Point 3

*Patient involvement in shared decision-making should be encouraged.*

Paternalistic medicine has long had its day. Guidelines and expert opinion statements on hypertension treatments have long acknowledged the individual responsibility for BP management and the overall importance of patient involvement. Treatment decisions should be taken together between physicians and patients and include the generation of personal risk profiles, as well as an informed discussion of patient preferences, motivation and treatment expectations, including patient tolerance for risk and perspective on benefit [82]. One problem is that standardised patient-reported outcomes (PRO) measures are lacking for uncontrolled hypertension treatment, whether with medications alone, or following an intervention such as RDN [4]. Important research is being carried out on patient preferences and potential concerns with interventional therapies [83,84,85]. In recent surveys, approximately one-third of hypertensive patients preferred RDN over escalation of medications for BP control [83,84]. Treatment-related risks seem to have less influence on treatment choices than the potential for benefits [85]. Yet it remains unclear to date whether BP reduction represents patients’ highest priority or if, e.g., stroke avoidance or pill reduction may be seen as more important. In a recent study, adults with hypertension in the United States put greater importance on a reduction in cardiac risk than on adverse events related to antihypertensive therapy. More work is clearly needed, particularly around interventional therapies.

### 8.4. Point 4

*Renal denervation is effective at lowering BP and appears safe.*

The guiding rule when deciding on the appropriateness of an intervention is that the procedural risk must not exceed the underlying risk of the untreated condition for each individual patient. As was discussed in detail above, this is considered well demonstrated for RDN in hypertension. 

### 8.5. Point 5

*Renal denervation should prioritise patients with uncontrolled hypertension and elevated CV risk and those with established organ damage.*

RDN was initially trialled in patients with resistant hypertension, but there are several reasons why this may not be the most appropriate target group. The term ‘resistant hypertension’ is overused and different guidelines define it in different ways. US guidelines recommend to limit its application to subjects with office SBP/DBP ≥ 130/80 mm Hg, despite prescription of ≥ three antihypertensive medications at optimal doses, including a diuretic if possible or a requirement of ≥ four antihypertensive medications to achieve an office SBP/DBP < 130/80 mmHg (diastolic BP = DBP) [86]. ESC guidelines define resistant hypertension when the recommended treatment strategy (lifestyle measures and optimal or best-tolerated doses of ≥ three antihypertensive drugs which should include one diuretic) fails to lower office SBP and DBP values to <140 mmHg and/or <90 mmHg, respectively, and the inadequate control of BP is confirmed by ambulatory or home BP measurement [8]. 

European and US consensus statements on RDN prioritise patients with uncontrolled hypertension and elevated CV risk, possibly with established CV event or organ damage [4,13,16]. With focus on patients with Asian ethnicity, the Asian Renal Denervation Consortium extends its recommendations for RDN not only to patients with resistant hypertension, but also as a treatment to be considered in patients with uncontrolled hypertension in the context of established atherosclerotic disease [14]. RDN is associated with a BP reduction that can match that of a dual-agent combination for some patients [87]. This makes it an appealing complementary treatment option in high-risk individuals with a high burden of polypharmacy and/or high CV risk.

### 8.6. Point 6

*Centres performing renal denervation should asses efficacy and safety endpoints, as well as follow-up data in patients undergoing renal denervation in standardised and auditable databases.*

Office BP measurements as well as ABPM should be considered as standard of care to assess the efficacy of treatment post RDN. The optimal timepoint for follow-up is yet to be defined in the context of a progressive treatment effect over time as published in the trials. The collection of data is imperative to grant high procedural standards and optimise patient selection and outcomes. It allows auditing the efficacy and safety of a novel treatment and identifying any potential futility of efficacy in real-world patients.

## 9. Open Questions

The long-term effects of RDN will become evident as registries continue to follow up treated patients. In the context of the non-responder (a < 5 mmHg reduction in SBP after RDN) rates in the absence of antihypertensive drugs, 37% in SPYRAL HTN-OFF MED and 36% in RADIANCE HTN-Solo, there is an unmet need to identify predictors of response [9,11]. All RCTs reported a large variation in BP reductions with RDN treatment. In RADIANCE HTN-Solo, changes in daytime ambulatory SBP at 2 months varied by over 30 mm Hg [11]. 

Variability in baseline BP levels and changes in medication or adherence during follow-up make it very difficult to identify true responders in a study population [47,48]. Post hoc analysis of the SPYRAL HTN-ON MED data suggested an elevated ambulatory heart rate (≥73.5 bpm (cohort median)) to be correlated with response to RDN, with an elevated heart rate being a surrogate marker for sympathetic activity [88]. However, in RADIANCE HTN-Solo, this arbitrary cut-off (representing the median value in the SPYRAL HTN-ON MED trial) had an insufficient predictive value [89]. In this trial, level and variability of night-time SBP seemed to predict response, but with low sensitivity [89]. 

At the time of writing, only the level of BP at baseline has been confirmed to be a reliable indicator of response. However, this goes for most antihypertensive treatments and may be in part due to Wilder’s law of initial value, which states that the direction of response of body function to any agent depends to a large degree on the initial value of that function [90]. In the SPYRAL HTN-OFF MED trial, elevated PRA was associated with greater effects from RDN on 24 h SBP reduction, making PRA a potential predictor of response to RDN [91].

Procedural success has been reported to drive the response to RDN: in SYMPLICITY HTN-3, insufficient denervation was one of the factors driving the neutral outcome [47]. Post hoc uni- and multivariate regression analyses of the RADIANCE HTN-Solo data found daytime ambulatory DBP and antihypertensive medication at the moment of screening to be potential predictors of response to ultrasound-based RDN [92]. Although underpowered for the list of tested predictors, orthostatic hypertension as a marker of increased sympathetic activity as well as the presence of untreated accessory arteries were predictors of response to RDN in the univariate analysis and should be part of further investigations [92]. In animal models, SBP elevation after renal nerve stimulation was used to identify the most responsive ablation sites [93]. As proof of feasibility studies in humans, this concept of high-frequency electrical stimulation of renal artery nerves before and after RDN has been applied to identify potential predictors of response (such as blunted SBP response or changes in heart rate variability after RDN) [94,95]. However, it is uncertain whether this method can or should be adapted into routine clinical practice.

Although CV outcome-driven clinical trials for RDN are pending, the effect size achieved with RDN and the proven MACE reductions after medication-induced BP reductions, as well as the probabilistic models of MACE reductions in the Global SYMPLICITY Registry give no reasons to doubt the potential for MACE reductions after RDN-based BP control [3,67,68].

## 10. Conclusions

It is our opinion that RDN should be considered an important emerging tool in the management of hypertension. The therapeutic approach needs to be multidisciplinary and personalised to take into account the perspective of patients and healthcare professionals in a shared decision-making process. Important questions regarding the identification of responders, as well as long-term efficacy and safety of the intervention are to be met. But today’s clinical results and experience with the intervention should reassure physicians and patients that RDN can provide much-needed help to address hypertension and its adverse consequences. For now, the use of RDN in Ireland should be limited within research registries and centres of excellence. Though in the future, as more evidence for efficacy and cost-effectiveness emerges to inform changes to hypertension clinical practice guidelines, it is hoped that RDN will become an option for all patients and healthcare providers to consider.

## Supplementary Materials

The following supporting information can be downloaded at: https://www.mdpi.com/article/10.3390/jcm12175634/s1, Table S1: Blood pressure cut-offs used for trial inclusion in the different trials and kidney level exclusion criteria. References [96,97] are cited in the supplementary materials.
