# Supplementary material for: Renal Denervation as a Complementary Treatment Option for Uncontrolled Arterial Hypertension: A Situation Assessment
Source: J Clin Med. 2023 Aug 29;12(17):5634. doi: 10.3390/jcm12175634 (PMC10488551; doi:10.3390/jcm12175634)
Supplement: Supplementary file 1 [file jcm-12-05634-s001.zip › jcm-2547359-supplementary.pdf]

Supplementary Appendix

Renal denervation a complementary treatment option for uncontrolled arterial hypertension. A situation assessment.

Max Wagener [1,†], Eamon Dolan [2,†], Samer Arnous [3], Joseph Galvin [4], Andrew W Murphy [5], Ivan Casserly [4], Joseph Eustace [6], Stephen O'Connor [7], Charles McCreery [8], James Shand [8], Catherine Wall [9], Saijad Matiullah [10] and Faisal Sharif [1,*]

[1] University Hospital Galway, University of Galway, H91 TK33 Galway, Ireland; max.wagener@gmail.com
[2] Stroke and Hypertension Unit, Connolly Hospital, D15 X40D Dublin, Ireland; eamon.dolan@hse.ie
[3] University Hospital Limerick, V94 F858 Limerick, Ireland; samer.arnous@hse.ie
[4] The Mater Misericordiae University Hospital, D07 R2WY Dublin, Ireland; secdrjosephgalvin@mater.ie (J.G.); secdrivancasserly@mater.ie (I.C.)
[5] Turloughmore Medical Centre, University of Galway, H91 TK33 Galway, Ireland; andrew.murphy@nuigalway.ie
[6] Health Research Board Clinical Research Facility, University College Cork, T12 K8AF Cork, Ireland; j.eustace@ucc.ie
[7] St. James's Hospital, D08 NHY1 Dublin, Ireland; soconnorsec@stjames.ie
[8] St. Vincent's University Hospital, D04 T6F4 Dublin, Ireland; charles.mccreery@svhg.ie (C.M.); jamesshand@svhg.ie (J.S.)
[9] Tallaght University Hospital, D24 NR0A Dublin, Ireland; catherine.wall@tuh.ie
[10] University Hospital Waterford, X91 ER8E Waterford, Ireland; sajjad.matiullah@hse.ie
* Correspondence: faisal.sharif@universityofgalway.ie; Tel.: +353-86-1455568
† These authors contributed equally to this work.

Trial	BP cut-offs for inclusion	Kidney level exclusion criteria
SPYRAL HTN-OFF/ON MED/OFF MED Pivotal	**office BP:**<br>SBP ≥ 150 mmHg and < 180mmHg and DBP ≥ 90 mmHg<br><br>**and**<br><br>**24h-ABPM:**<br>SBP ≥ 140 mmHg and < 170 mmHg	**anatomic:**<br>Absence of at least 1 renal artery > 3 mm and < 8 mm<br>Main renal artery stenosis > 50%<br>Renal artery segments containing atheroma, aneurysm, fibromuscular dysplasia, or calcification<br>Renal artery stent within 3 months or treatment within 5 mm of a stent in place > 3 months<br>Single kidney<br><br>**function:** eGFR < 45 mL/min/1.73 m2
RADIANCE Solo/Trio/II	**office BP (screening):** ≥ 140/90 mmHg and <180/110 mmHg<br><br>**daytime ABPM:**<br>≥ 135/85 mmHg (and <170/105 mmHg in Solo and Radiance II Cohort)	**anatomic:**<br>Main renal artery diameter <4 (<3mm in Radiance II) or >8 mm, length <25 mm (<20 mm in Trio/Radiance II)<br>Single functioning kidney<br>Abnormal kidney (or secreting adrenal) tumors<br>Renal arerty aneurysm<br>Pre-esisting renal stent or history of renal artery angioplasty<br>Prior renal denervation procedure<br>Fibromuscular disease of renal arteries<br>Renal artery stenosis of any origin ≥30%<br>Accessory renal artery diameter ≥2 and <4 mm (<3mm in Trio/Radiance II) or >8 mm<br><br>**function:** eGFR < 40 mL/min/1.73 m2

Table S1 – Blood pressure cut-offs used for trial inclusion in the different trials and kidney level exclusion criteria. [1–9]

References

1. Townsend RR, Mahfoud F, Kandzari DE, Kario K, Pocock S, Weber MA, et al. Catheter-based renal denervation in patients with uncontrolled hypertension in the absence of antihypertensive medications (SPYRAL HTN-OFF MED): a randomised, sham-controlled, proof-of-concept trial. Lancet. 2017 Nov 11;390(10108):2160–70.

2. Azizi M, Schmieder RE, Mahfoud F, Weber MA, Daemen J, Davies J, et al. Endovascular ultrasound renal denervation to treat hypertension (RADIANCE-HTN SOLO): a multicentre, international, single-blind, randomised, sham-controlled trial. Lancet. 2018 Jun 9;391(10137):2335–45.

3. Kandzari DE, Böhm M, Mahfoud F, Townsend RR, Weber MA, Pocock S, et al. Effect of renal denervation on blood pressure in the presence of antihypertensive drugs: 6-month efficacy and safety results from the SPYRAL HTN-ON MED proof-of-concept randomised trial. Lancet. 2018 09;391(10137):2346–55.

4. Böhm M, Kario K, Kandzari DE, Mahfoud F, Weber MA, Schmieder RE, et al. Efficacy of catheter-based renal denervation in the absence of antihypertensive medications (SPYRAL HTN-OFF MED Pivotal): a multicentre, randomised, sham-controlled trial. Lancet Lond Engl. 2020 May 2;395(10234):1444–51.

5. Azizi M, Sanghvi K, Saxena M, Gosse P, Reilly JP, Levy T, et al. Ultrasound renal denervation for hypertension resistant to a triple medication pill (RADIANCE-HTN TRIO): a randomised, multicentre, single-blind, sham-controlled trial. Lancet Lond Engl. 2021 May 14;

6. Kario K, Mahfoud F, Kandzari DE, Townsend RR, Weber MA, Schmieder RE, et al. Long-term reduction in morning and nighttime blood pressure after renal denervation: 36-month results from SPYRAL HTN-ON MED trial. Hypertens Res Off J Jpn Soc Hypertens. 2023 Jan;46(1):280–8.

7. Kandzari DE, Kario K, Mahfoud F, Cohen SA, Pilcher G, Pocock S, et al. The SPYRAL HTN Global Clinical Trial Program: Rationale and design for studies of renal denervation in the absence (SPYRAL HTN OFF-MED) and presence (SPYRAL HTN ON-MED) of antihypertensive medications. Am Heart J. 2016 Jan;171(1):82–91.

8. Azizi M, Saxena M, Wang Y, Jenkins JS, Devireddy C, Rader F, et al. Endovascular Ultrasound Renal Denervation to Treat Hypertension: The RADIANCE II Randomized Clinical Trial. JAMA. 2023 Feb 28;329(8):651–61.

9. Mauri L, Kario K, Basile J, Daemen J, Davies J, Kirtane AJ, et al. A multinational clinical approach to assessing the effectiveness of catheter-based ultrasound renal denervation: The RADIANCE-HTN and REQUIRE clinical study designs. Am Heart J. 2018 Jan 1;195:115–29.
